# Enhanced articular cartilage regeneration with SIRT1-activated MSCs using gelatin-based hydrogel

**DOI:** 10.1038/s41419-018-0914-1

**Published:** 2018-08-29

**Authors:** Seong Mi Choi, Kyoung-Mi Lee, Seung Bae Ryu, Yoo Jung Park, Yeok Gu Hwang, Dawoon Baek, Yoorim Choi, Kwang Hwan Park, Ki Dong Park, Jin Woo Lee

**Affiliations:** 1 Department of Orthopaedic Surgery, Yonsei University College of Medicine, 50-1 Yonsei-ro, Seodaemun-gu, Seoul, 03722 South Korea; 2 Brain Korea 21 PLUS Project for Medical Sciences, Yonsei University College of Medicine, 50-1 Yonsei-ro, Seodaemun-gu, Seoul, 03722 South Korea; 3 Severance Biomedical Science Institute, Yonsei University College of Medicine, 50-1 Yonsei-ro, Seodaemun-gu, Seoul, 03722 South Korea; 4 Department of Molecular Science and Technology/Applied Chemistry and Biological Engineering, Ajou University, 206, World cup-ro Yeongtong-gu, Suwon-si, Gyeonggi-do 16499 South Korea; 5 Department of Orthopaedic Surgery, Yonsei University Wonju College of Medicine, 20, Ilsan-ro, Wonju-si, Gangwon-do 26426 South Korea

## Abstract

To investigate the functional effects of resveratrol (RSV) on mesenchymal stem cells (MSCs), we treated MSCs with RSV continuously during ex vivo expansion. MSCs were continuously treated with RSV from passage (P) 0 to P5. A proliferative capacity of RSV-treated MSCs was higher than that of non-treated MSCs and similar with P1-MSCs. Continuous treatment of RSV on MSCs increased the stemness and inhibited the senescence. During chondrogenic differentiation in vitro, RSV-treated MSCs had higher differentiation potential and reduced hypertrophic maturation, which are limitations for hyaline cartilage formation. The histological analysis of micromass demonstrated increased chondrogenic differentiation potential. We further explored the therapeutic effectiveness of this method in a rabbit osteochondral defect model. A rabbit osteochondral defect model was established to investigate the hyaline cartilage regeneration potential of RSV-treated MSCs. Moreover, the cartilage regeneration potential of RSV-treated MSCs was greater than that of untreated MSCs. The expression levels of chondrogenic markers increased and those of hypertrophic markers decreased in RSV-treated MSCs compared with untreated MSCs. Sustained treatment of RSV on MSCs during ex vivo expansion resulted in the maintenance of stemness and enhanced chondrogenic differentiation potential. Consequentially, highly efficient MSCs promoted superior hyaline cartilage regeneration in vivo. This novel treatment method provides a basis for cell-based tissue engineering.

## Introduction

Osteoarthritis (OA) involves cartilage damage, dysfunctional chondrocyte proliferation, and hypertrophic maturation^1–4^. Several types of therapies are currently used for cartilage regeneration, including bone marrow-stimulating techniques, mosaicplasty, and cell-based therapies^5^. Cell-based therapies are increasingly used as a prospective treatment. Autologous chondrocyte implantation is commonly used for cartilage regeneration, requiring the in vitro expansion of autologous chondrocytes^6^. Nevertheless, these techniques have several shortcomings, such as their complexity, cost, and the loss of cartilage capacity^7,8^. Therefore, cell-based therapeutic approaches using mesenchymal stem cells (MSCs) have emerged for cartilage regeneration^9–11^.

MSCs have various advantages for clinical applications, such as anti-inflammatory and immunosuppressive effects, high effectiveness, and a lack of severe side effects^12^. Additionally, MSCs possess a high stemness capacity and thereby have the potential for multipotency, including chondrogenic differentiation. For these reasons, MSCs are a promising cell source for cartilage regeneration^13–15^. However, several factors limit the clinical application of MSCs. For example, hundreds of millions of MSCs are required. MSCs can be isolated from various organs, but the number of isolated cells is insufficient. To obtain sufficient MSCs, long-term in vitro expansion is necessary^16^; however, acquiring an effective quantity of MSCs with sustained self-renewal and multi-lineage differentiation potential is difficult^17,18^. Moreover, MSCs tend to become fibro-like tissues after the induction of chondrogenic differentiation^19,20^, and they exhibit fibro-like tissue regeneration in vivo. Therefore, the identification of new environments for the development of highly efficient MSCs with an enhanced self-renewal capacity and chondrogenic differentiation potential is necessary.

Mohyeldin et al.^21^ demonstrated that MSCs require physiological oxygen levels of between 2 and 8%. Under hypoxic conditions, the expression levels of hypoxia-inducible factors increase, leading to the upregulation of Oct-4, Sox2, and Nanog, which are critical transcription factor for stemness^22^. Previous studies have suggested that under hypoxic conditions, MSCs have enhanced proliferative potential and stemness during in vitro cultivation^23^. In fact, antioxidants, such as resveratrol^24^, palm vitamin E^25^, butylated hydroxyanisole^26^, and butylated hydroxytoluene^27^, are known to not only suppress reactive oxygen species, but also delay disease progression and influence lipid peroxidation^28^. Butylated hydroxyanisole and butylated hydroxytoluene are frequently used as additives in the food industry^26,29^; however, their use is restricted owing to their potential toxicity and carcinogenic effects^28^. Thus, the discovery of new, safe antioxidants that can overcome the disadvantages of chemical antioxidants is necessary.

The antioxidant resveratrol (RSV; 3,5,4ʹ-hydroxystilbene), a natural compound, is a phytoalexin produced by plants in response to environmental stress^30–32^. RSV has critical roles in the inhibition of oxidative damage^33^ as well as in cell survival and proliferation^34–36^. Furthermore, RSV is known to enhance activity of sirtuin 1 (SIRT1), an NAD+-dependent lysine deacetylase. During in vitro cultivation, MSCs occasionally become senescent, leading to a loss of stemness. Previously, Yuan et al.^35^ demonstrated that the knockdown of SIRT1 in early-passage MSCs using shRNA results in a loss of proliferative capacity and promotes cellular senescence. In contrast, the overexpression of SIRT1 in MSCs delays cellular senescence and cells maintain multipotency during long-term in vitro cultivation^35^. Moreover, RSV enhances the osteogenic and adipogenic differentiation potential of MSCs, but prolonged treatment of MSCs with RSV induces senescence^37,38^. Other studies have demonstrated that RSV has negative effects on MSCs during adipogenic and osteogenic differentiation^39,40^. These incompatible results regarding the effects of RSV on senescence and differentiation in MSCs may be explained by dose- or duration-dependent effects^34^. Hence, a novel RSV treatment method for MSCs is necessary. Additionally, the effect of RSV on chondrogenic differentiation has not been elucidated. In this study, we developed a novel treatment method using RSV that yields consistent results. Moreover, we evaluated the stemness and chondrogenic differentiation potential of MSCs after treatment with RSV. This novel treatment method provides a basis for cell-based tissue engineering.

## Results

### Increased stemness and decreased senescence of RSV-treated MSCs

**Fig. 1:**
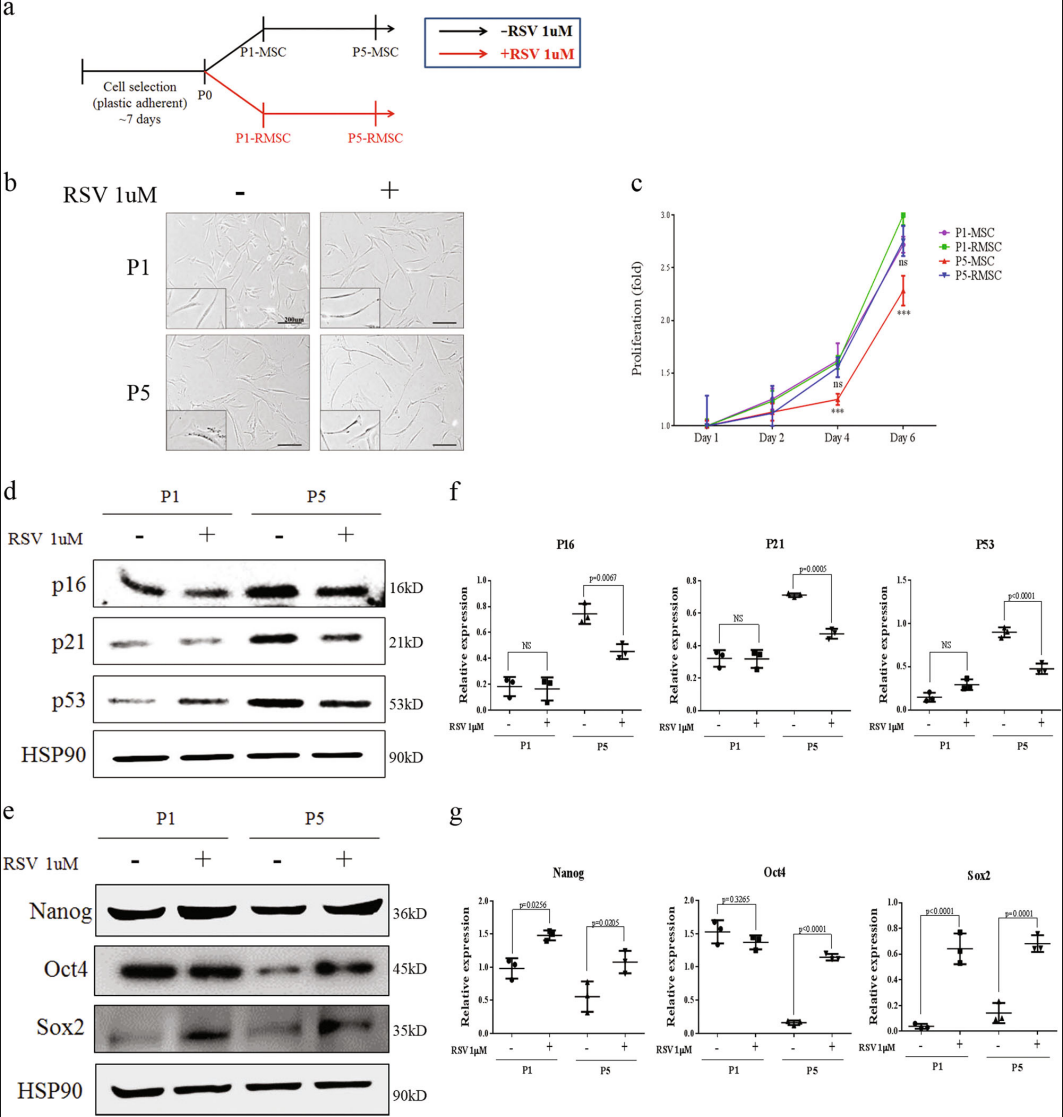
Increased stemness of MSCs during continuous treatment with RSV. **a** Schematic overview of RSV treatment. **b** Morphological changes of MSCs with passaging. Comparison between MSCs treated with RSV and without RSV (bar = 200 µm). **c** Proliferation, as determined on day 1, 2, 4, and 6. Expression levels of senescence markers, P16, P21, and P53 (**d**) and stemness markers, Nanog, Oct4 and Sox2 (**e**) in P1-MSC, P1-RMSC, P5-MSC, and P5-RMSC, determined by western blotting. Quantitative analysis of protein expression levels of senescence (**f**) and stemenss (**g**) markers was performed. Each dot represents a mean value of triplicate estimates with one donor and horizontal lines display the means and 95% CIs of the values from different donors per group

During in vitro cultivation, we continuously treated MSCs with RSV from P0 to P5 (P5-RMSC), and other cells were cultured up to P5 without RSV treatment (P5-MSC) (Fig. 1a). To investigate whether RSV treatment increases the self-renewal capacity of MSCs, we evaluated the cell morphology and proliferative potential. P5-RMSCs had a similar morphology to that of P1-MSCs, i.e., cells were small and spindle-shaped. However, P5-MSCs had a large, flat appearance (Fig. 1b). P5-MSCs had a decreased proliferative capacity over time, whereas the P5-RMSCs had a similar proliferative capacity to that of P1-MSCs (Fig. 1c). To determine the effects of RSV on senescence and stemness, we assessed protein expression levels of senescence and stemness markers such as P16, P21, P53 and Nanog, Oct4, Sox2, respectively. In P5-RMSCs, the stemness markers were up-regulated and senescence markers were down-regulated, similar to the expression levels in P1-MSCs, however, the opposite results were obtained for P5-MSCs, which exhibited the upregulation of senescence markers and down-regulation of stemness markers (Fig. 1d, e). Therefore, continuous treatment of RSV on MSCs was able to maintain the stemness and inhibit senescence, suggesting that it is a useful method for the establishment of highly efficient MSCs.

### Enhancement of the chondrogenic differentiation potential in RSV-treated MSCs

**Fig. 2:**
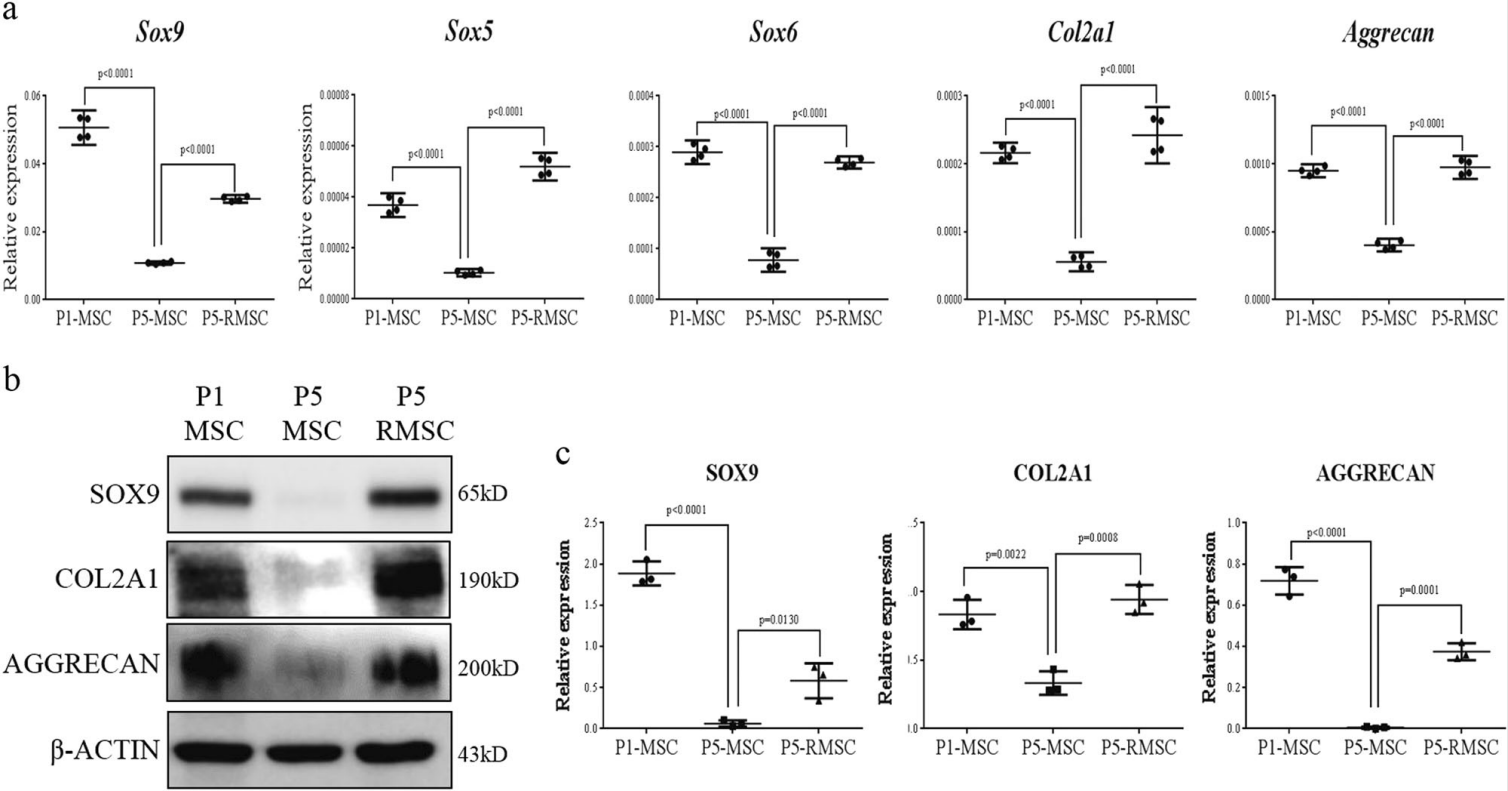
Enhanced chondrogenic differentiation of P5-RMSC *in vitro*. **a** Relative expression levels of chondrogenic differentiation markers by real-time PCR after chondrogenic differentiation on day 5. β-actin was used as an internal control. Western blotting was used to evaluate the chondrogenic markers sox9, col2a1, and aggrecan (**b**) and a quantitative analysis of protein expression levels was performed (**c**). Each dot represents a mean value of triplicate estimates with one donor and horizontal lines display the means and 95% CIs of the values from different donors per group. *P*-values were calculated for comparisons with P5-MSC

**Fig. 3:**
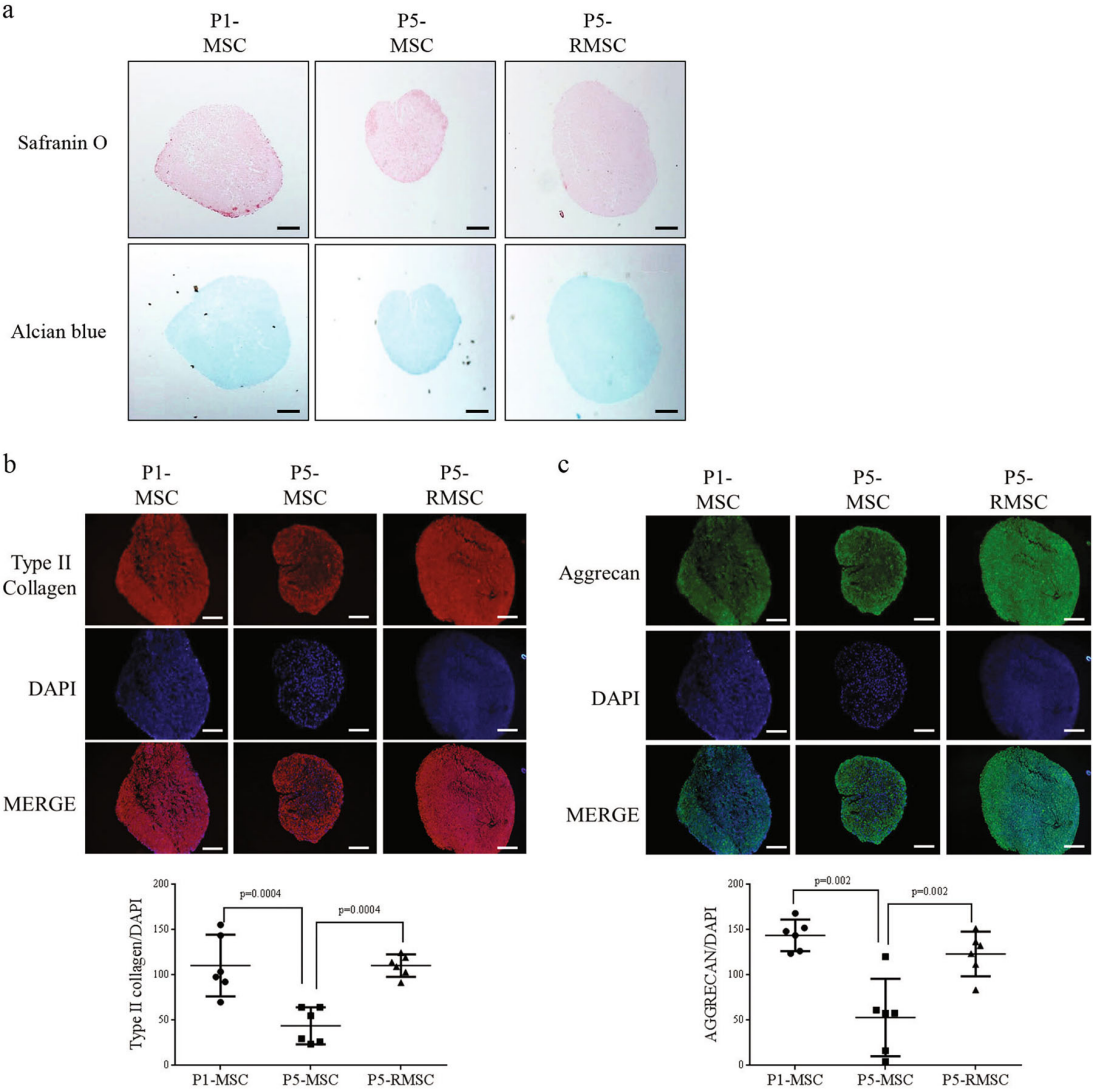
Histological analysis of chondrogenic differentiation in vitro. **a** Safranin O and Alcian blue staining of micromass cultured MSCs on day 14 (bar = 200 µm). **b**, **c** Immunofluorescence analyses of type II collagen and aggrecan, chondrogenic markers, were performed (upper) and results were quantitatively analyzed (bottom) (bar = 100 µm). Each dot indicates a mean and 95% CI. *P*-values were calculated for comparisons with P5-MSC (*n* = 3, triplicates for each donor)

To evaluate the chondrogenic differentiation potential, we performed micromass culture using P1-MSC, P5-MSC, and P5-RMSC. The mRNA levels of *Sox-5, -6, -9, Col2a1*, and *aggrecan*, which are chondrogenic markers, were higher in P5-RMSC than in P5-MSC and were similar to the levels in P1-MSC (Fig. 2a). The protein expression levels of chondrogenic markers were comparable in P1-MSC and P5-RMSC and a quantitative analysis supported this finding (Fig. 2b, c). To further demonstrate the increased chondrogenic differentiation potential of P5-RMSC, we performed SafO and AB staining. The P5-MSC group had a much smaller micromass size than those of the P1-MSC and P5-RMSC groups, which both had higher contents of glycosaminoglycan (GAG) and proteoglycan (PG) (Fig. 3a). We evaluated the expression levels of *col2a1* and aggrecan in micromass pellets by immunocytochemistry. P5-RMSC had high expression levels of *col2a1* and aggrecan, similar to the expression levels in P1-MSC (Fig. 3b, c). Accordingly, P5-RMSC, which had enhanced stemness, had superior chondrogenic differentiation potential compared to that of P5-MSC.

### Inhibition of the hypertrophic maturation of RSV-treated MSCs

**Fig. 4:**
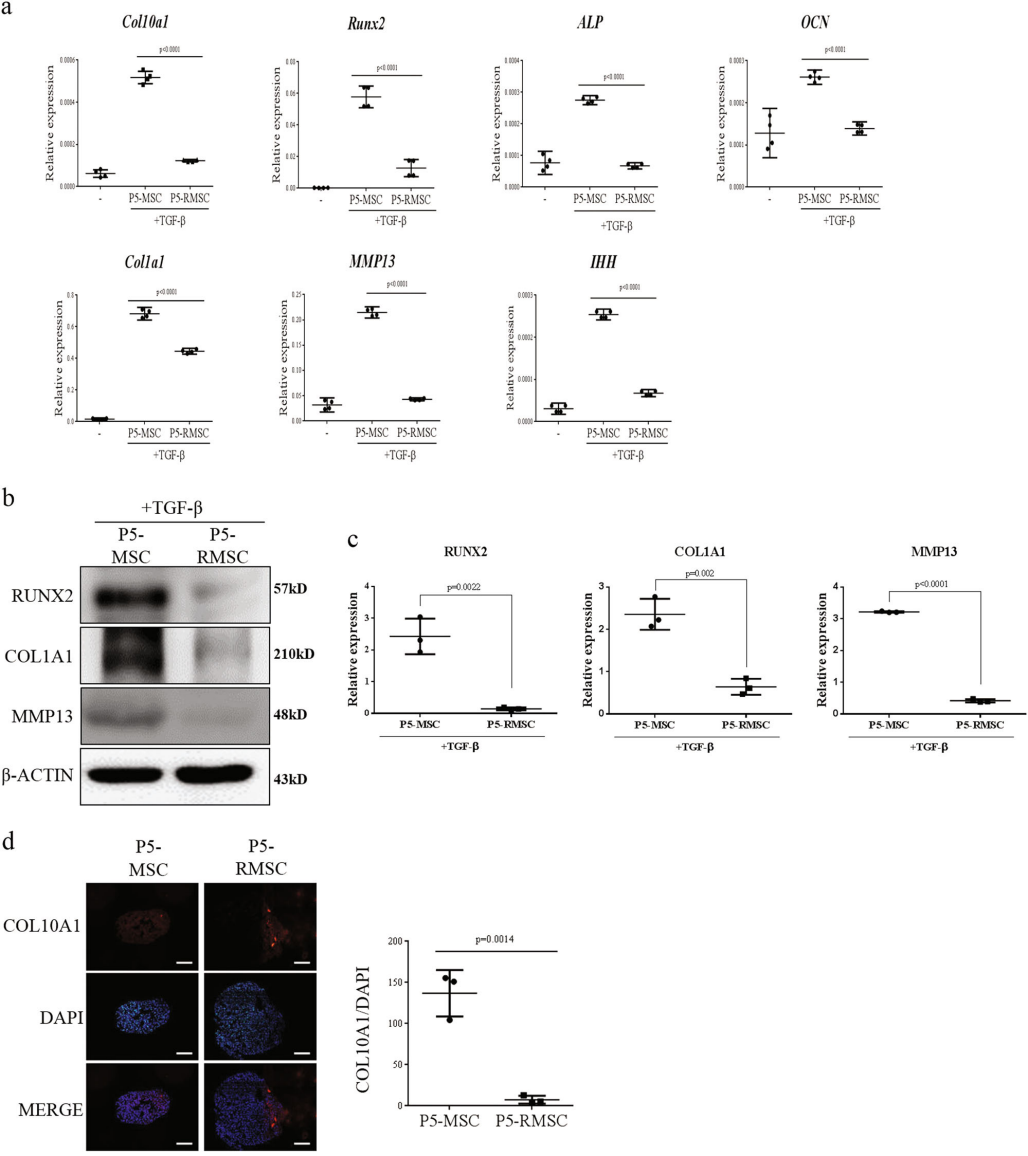
Inhibition of hypertrophic maturation during chondrogenic differentiation. **a** Relative expression levels of hypertrophic markers, as determined by real-time PCR, after chondrogenic differentiation on day 21. **b**, **c** Hypertrophic markers were analyzed by western blotting on day 21 and quantitative analysis of protein expression levels was performed. **d** Immunofluorescence analysis of col10a1, a hypertrophic maturation marker, on day 21 (left) and quantification of col10a1 expression (right) (bar = 100 µm). Each dot indicates a mean and 95% CI. *P*-values were calculated for comparisons with P5-MSC (*n* = 3, triplicates for each donor)

During chondrogenic differentiation, hypertrophic maturation inhibits hyaline cartilage regeneration^19,41^. To determine whether P5-RMSC exhibited inhibited hypertrophic maturation during chondrogenic differentiation in vitro, we evaluated the both mRNA and protein expression levels of hypertrophic markers. P5-RMSC had lower expression levels of hypertrophic markers than those in P5-MSC (Fig. 4a, b). Additionally, an immunocytochemical analysis demonstrated that the expression of col10a1, a key marker of hypertrophic maturation, was significantly decreased in P5-RMSC (Fig. 4c). Consequentially, sustained treatment of MSCs with RSV enhanced the chondrogenic differentiation potential and prevented hypertrophic maturation during in vitro chondrogenic differentiation via improved stemness.

### Increased cartilage regeneration potential in vivo

**Fig. 5:**
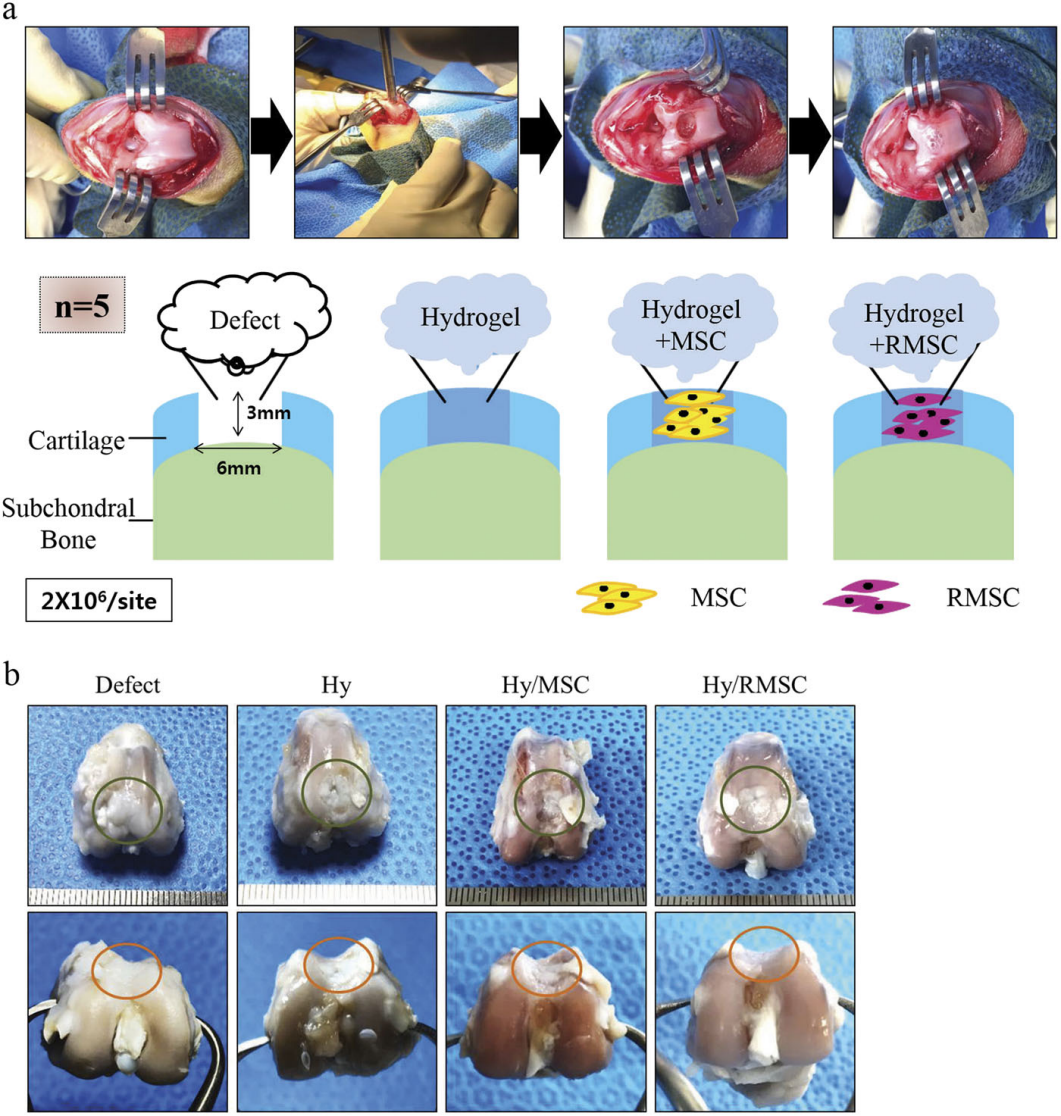
Regenerative potential of cartilage in a rabbit osteochondral defect model. **a** Establishment of the rabbit osteochondral defect model. A total of 2 × 10^6^ cells were applied to osteochondral defect sites (diameter, 6 mm; depth, 3 mm) with a hydrogel scaffold (*n* = 5). **b** Gross morphology of osteochondral defect sites after 8 weeks

To determine whether continuous treatment of RSV on MSCs could enhance the cartilage regeneration capacity in vivo, we established a rabbit osteochondral defect model (Fig. 5a). At 8 weeks post operation, we observed the gross morphology of regenerated cartilage. In the Hy/RMSC group, the surface of the defect site was almost fully filled with cartilage-like tissue, along with nearby cartilage. The Hy/RMSC group showed transparent cartilage-like tissues, whereas the defect, Hy, and Hy/MSC groups had incomplete cartilage tissue formation (Fig. 5b). These results indicate that the regeneration of intact cartilage surface tissues is possible via continuous treatment of RSV on MSCs.

### Histological analysis of regenerated cartilage in the rabbit osteochondral defect model

**Fig. 6:**
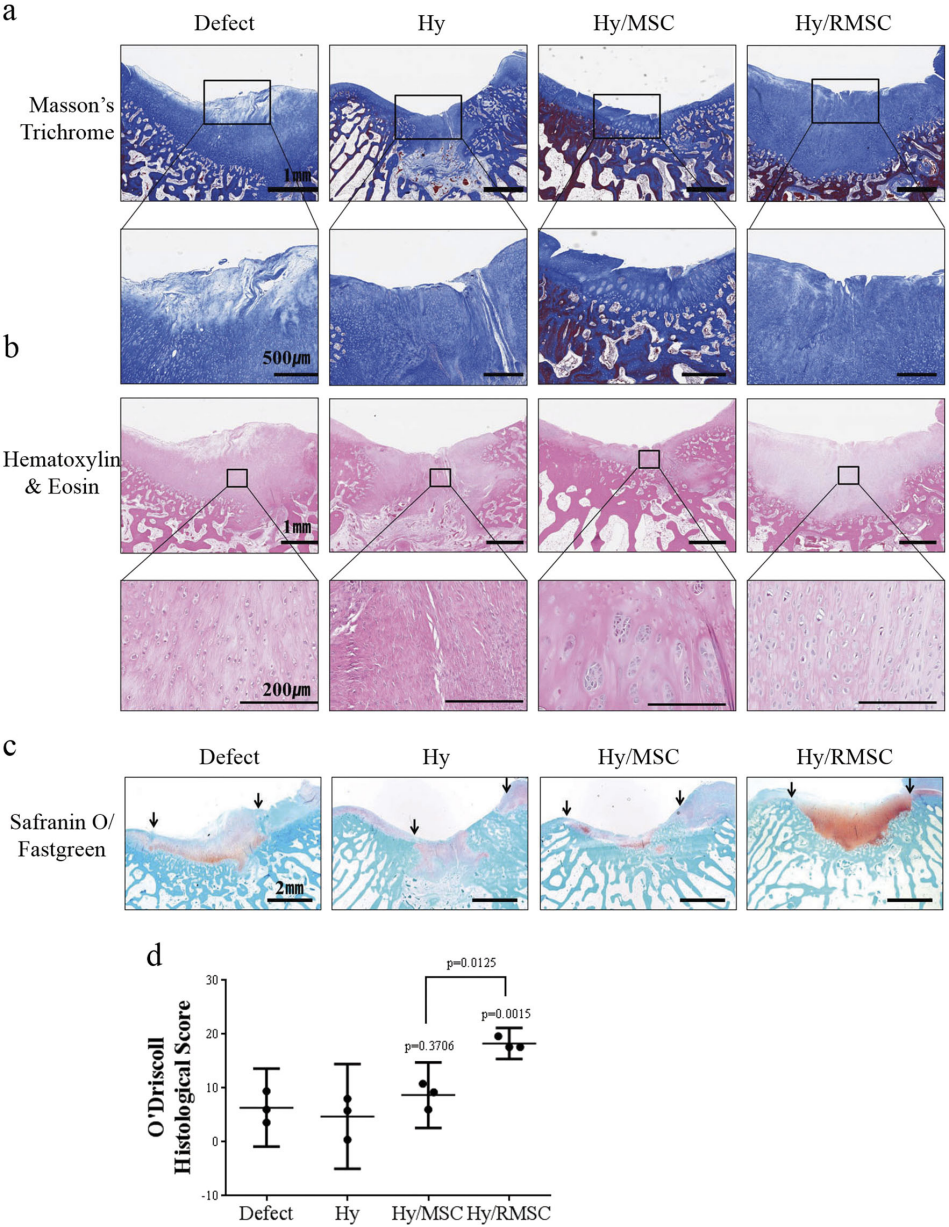
Histological examination of osteochondral defect sites. **a**, **b** Representative MT and H&E staining results for osteochondral defect sites of each group at 8 weeks post operation. **a** Collagen deposition at osteochondral defect sites (upper; bar = 1 mm) and enlarged images to confirm collagen fiber alignment (bottom; bar = 500 µm). Blue area indicates newly formed collagen tissues. **b** H&E staining shows the cell morphology at osteochondral defect sites (upper; bar = 1 mm, bottom; bar = 200 µm). **c** Safranin O/Fast Green staining indicates newly formed cartilage tissues (bar = 2 mm). **d** Quantitative histological examination of regenerated cartilage at osteochondral defect sites. Three independent experts assessed the regenerated cartilage tissues (*n* = 3). *P*-values were calculated for comparisons with the defect group

We observed regenerated cartilage at defect sites by MT and H&E staining. The MT stain showed higher collagen deposition on cartilage surface of the Hy/RMSC group compared to the Hy/MSC group (Fig. 6a). In the H&E stain, the Hy/RMSC group had chondrocyte-like cells with smooth cartilage-like tissues, which were similar in morphology to normal cartilage. However, the defect, Hy, and Hy/MSC groups showed fibrous tissue formation (Fig. 6b). Safranin O/Fast Green staining demonstrated increased GAG formation in the Hy/RMSC group; the Hy/MSC group showed slightly greater synthesis of GAG than those of the other groups (Fig. 6c). Additionally, O’Driscoll histological scoring was performed, and the Hy/MSC group had a slightly higher score than those of the defect or Hy group, and the Hy/RMSC group had a significantly higher score (Fig. 6d). These results demonstrate that the Hy/MSC group had slight differences in cartilage regeneration, but Hy/RMSC group exhibited significant differences in functional cartilage regeneration.

### Effects of cartilage regeneration for RSV-treated MSCs based on both chondrogenic and hypertrophic markers

**Fig. 7:**
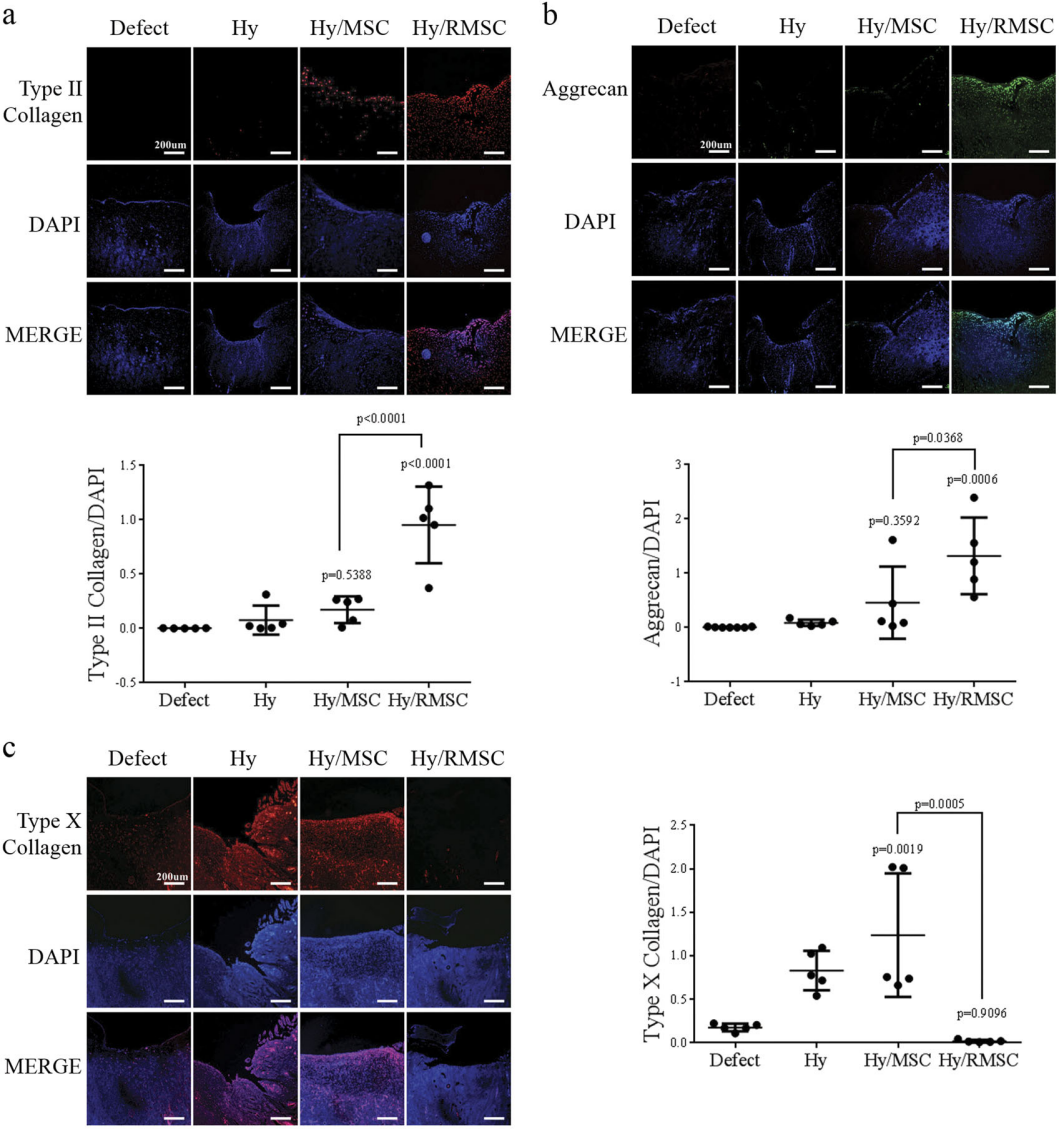
Immunofluorescence analysis of chondrogenic and hypertrophic markers. Representative images of type II collagen (**a**) and aggrecan (**b**), ECM proteins, in osteochondral defect sites (upper) and a quantitative analysis of chondrogenic marker expression (bottom). (**c**) Expression of type X collagen, a hypertrophic marker, in osteochondral defect sites (left) and a quantitative analysis of type X collagen levels (right). *P*-values were calculated for comparisons with the defect group (Bar = 200 µm)

Next, we evaluated the expression of extracellular matrix (ECM) proteins, type II collagen and aggrecan, in regenerated cartilage tissues by immunohistochemistry. The Hy/RMSC group showed higher expression levels of ECM proteins than those of the Hy/MSC group, which exhibited slight expression of these proteins. A quantitative analysis demonstrated that there was significantly higher expression of both type II collagen and aggrecan in the Hy/RMSC group than in other groups (Fig. 7a, b). Moreover, the expression of a hypertrophic marker, type X collagen, was assessed. The Hy/RMSC group showed very low expression of type X collagen, while other groups had high expression levels. A quantitative analysis showed that type X collagen expression was significantly lower in the Hy/RMSC group than in other groups (Fig. 7c). As a result, the continuous treatment of MSCs with RSV resulted in enhanced cartilage regeneration potential and the inhibition of hypertrophic maturation, which limits the use of MSCs for cell therapy^19,41^.

## Discussion

In the present study, MSCs were continuously treated with RSV from P0 to P5, and the maintenance of their stemness was confirmed. Continuous treatment maintained the activity of sirtuin 1 (SIRT1). P5-RMSC had increased protein levels of stemness markers and decreased levels of senescence markers compared with those in P5-MSC. As shown in our previous study, the maintenance of SIRT1 activity sustains the expression of SOX2^37^; thus, the stemness of MSCs could be maintained over time.

**Fig. 8:**
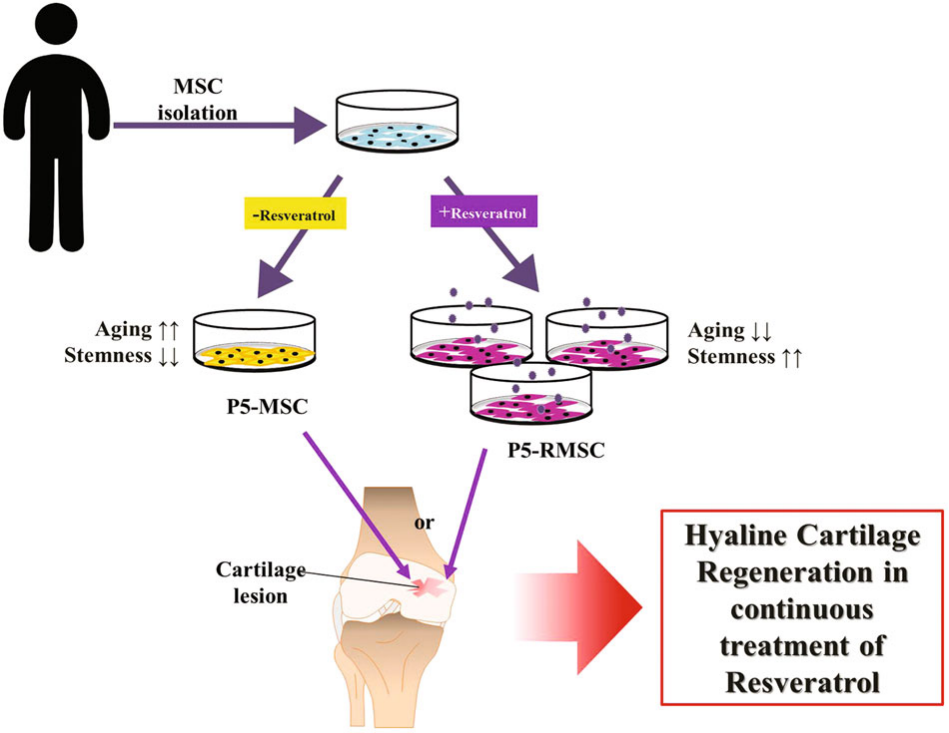
Schematic illustration of the enhanced cartilage regeneration potential in RSV-treated MSCs. Sustained treatment of MSCs with RSV from P0 to P5 increased stemness and inhibited senescence. With increased stemness, P5-RMSC had improved hyaline cartilage regeneration potential

P5-RMSC had similar stemness to that of P1-MSC and improved chondrogenic differentiation potential (Figs. 2 and 3). Additionally, hypertrophic maturation was inhibited in P5-RMSC, resulting in the increased expression of type X collagen  (COL10A1), matrix metalloproteinase 13 (MMP13), and alkaline phosphatase (ALP) (Fig. 4)^19,41^. To confirm the efficiency of the cartilage regenerative potential of P5-RMSC, which had similar stemness to that of P1-MSC, we established an osteochondral defect model in rabbits with a depth of 3 mm and a diameter of 6 mm, a critical size threshold for rabbit models^42^. This critical size of osteochondral defect sites results in an inability to self-heal. At 8 weeks post operation, P5-RMSC showed smoother and more intact cartilage that was well attached to nearby cartilage compared to other groups (Figs. 5 and 6). P5-RMSC had higher expression of ECM proteins and lower expression of hypertrophic maturation markers (Fig. 7). Therefore, P5-RMSC had increased cartilage regeneration potential with hyaline-like tissue formation in the rabbit osteochondral defect model when compared with P5-MSC. These results demonstrate that our innovative treatment method with RSV promotes the maintenance of stemness in MSCs over time and thus can increase the cartilage regeneration potential (Fig. 8).

During long-term ex vivo expansion, MSCs do not grow infinitely, leading to senescence^43^. Bonab et al.^17^ demonstrated that bone marrow-derived MSCs exhibit decreased population doubling and shorter telomere lengths as cells are subcultured. Furthermore, clinical history, age, and genetic makeup determine the length of the expansion period and quality of MSCs^44^. Thus, retaining MSCs with sustained stemness is essential.

Several studies have demonstrated that long-term exposure to RSV could increase the senescence of MSCs^34^; during osteogenic differentiation, RSV adversely affects adipogenic differentiation^45^. For the chondrogenic differentiation of MSCs, previous studies have demonstrated that treatment with RSV during the chondrogenic differentiation period enhances chondrogenic potential and inhibits inflammatory and degradative processes in cartilage via the activation of SIRT1^46^. Additionally, the treatment of chondrocytes with RSV up-regulates SIRT1 expression, but leads to hypertrophic maturation^47^. Unlike previous studies, our results showed that stemness can be maintained in P5-MSC via continuous treatment with RSV, thus maintaining their chondrogenic differentiation potential. We also confirmed the inhibition of hypertrophic maturation both in vitro and in vivo. For clinical applications, the recommended number of passages for MSCs is 3–5^48,49^. In general, MSCs at passages 1–2 have high multipotency and MSCs at passages 4–5 have low multipotency and a loss of replicative potential^50,51^. Accordingly, in our study, we continuously treated MSCs with RSV from P0 to P5 to verify the sustained stemness of MSCs at passage 5.

In our in vivo study, the defect group showed slight regeneration of cartilage-like tissues (Fig. 6). Although the osteochondral defect sizes were fixed at 3 mm in depth, during the surgical procedure, the bone marrow concentrates affected the regeneration of cartilage-like tissues in the defect group. In fact, bone marrow concentrates containing a variety of heterogenous cells and a lack of MSCs are known to contribute to fibrocartilage formation, rather than hyaline cartilage^52^. Thus, the bone marrow concentrates did not affect the cartilage regenerative potential in the Hy/MSC and Hy/RMSC groups. The Hy/RMSC group had hyaline-like cartilage tissue formation (Figs. 6 and 7).

Taken together, the continuous treatment of RSV on MSCs during in vitro cultivation resulted in sustained stemness and chondrogenic differentiation potential, similar to that of P1-MSC, thereby improving hyaline cartilage regeneration (Fig. 8). Therefore, the novel treatment method with RSV could provide a foundation for studies in cell-based tissue engineering with minimal side effects.

## Materials and methods

### Isolation and identification of MSCs from human bone marrow aspirates

Bone marrow aspirates were acquired from the posterior iliac crest of 10 healthy donors after obtaining approval from the Institutional Review Board of Yonsei University College of Medicine. MSCs were isolated and cultivated as previously described^53^.

### Chemical treatment of MSCs

Resveratrol (RSV; Sigma, St. Louis, MO, USA) was dissolved in ethanol (EtOH) at 1 µM. RSV was continuously applied to MSCs from P0 to P5 (P5-RMSC) and cells were subcultured as previously described^54^. MSCs were evaluated up to P5 based on previous studies indicating that MSCs at passages 3–5 are optimal for clinical applications^48,49^.

### In vitro chondrogenic differentiation of MSCs

For the chondrogenic differentiation of MSCs, the micromass culture method was used, as previously reported^55^. Briefly, 10 µl of resuspended cells at a density of 1 × 10^5^ cells per well was added to the center of the wells in 24-well plates. Cells were allowed to attach for 2 h and chondrogenic medium was then added. The chondrogenic medium was replenished every 3 days.

### Quantitative real-time PCR (qRT-PCR)

Extraction of total RNA harvested from cells was performed using TRIzol (Invitrogen, Carlsbad, CA, USA) according to the manufacturer’s protocols. To synthesize cDNA, the Omniscript Reverse Transcription Kit (Qiagen, Hilden, Germany) was used for the reverse transcription of 2 μg of RNA. To conduct real-time PCR, 2×qPCRBIO SyGreen Mix (PCR Biosystems, London, UK) was used with 80 ng of cDNA. To quantify the relative expression levels of target genes, beta-actin (β-actin) was used as an internal control. Validated primers targeting *Sox9* (P232240), *IHH* (P101104), and *ALP* (P324388) were used. Other primers were designed, as shown in Table 1, and all primers were purchased from Bioneer (Daejeon, Korea). Real-time PCR was performed using the ABI7900 (Applied Biosystems, Carlsbad, CA, USA) in accordance with a previous report^55^.

**Table 1:** Primer sequences for real-time PCR

Target gene	Forward sequence (5ʹ–3ʹ)	Reverse sequence (5ʹ–3ʹ)
*β-ACTIN*	GTCCTCTCCCAAGTCCACACAG	GGGCACGAAGGCTCATCATTC
*SOX5*	AGCCCCACATAAAGCGTCCAAT	GGTCCTCCTCCTCCTCATCGTA
*SOX6*	AGCAGAGCCTGTGAAGTCC	GGTCCTCCTCCTCCTCATCGTA
*COL2A1*	GGCAATAGCAGGTTCACGTACA	CGATAACAGTCTTGCCCCACTT
*AGGRECAN*	CCTGGCCTGACATGGAGCTG	GGACTGGGGGAGACCTCGAA
*RUNX2*	CCCAGTATGAGAGTAGGTGTCC	GGGTAAGACTGGTCATAGGACC
*OSTEOCALCIN*	AGCAAAGGTGCAGCCTTTGT	CTTCACTACCTCGCTGCCCT
*MMP13*	GACGGGGTTTTGCCACACTG	ATTGGGTGTGGTGGCTCACG
*COL1A1*	GCCCTGCTGGAGAGGAAGGA	ATTGGGTGTGGTGGCTCACG
*COL10A1*	CCAGGACAGCCAGGCATCAA	ATTGGGTGTGGTGGCTCACG

### Western blot analysis

Cell pellets were lysed and quantified as previously reported^56^. The samples (10–30 μg of protein) were separated by 10% sodium dodecyl sulfate-polyacrylamide gel electrophoresis (SDS-PAGE) (Sigma) and transferred onto polyvinylidene difluoride (PVDF) membranes (Amersham Pharmacia, Escondido, CA, USA). Briefly, after blocking with 5% skim milk, membranes were incubated with primary anti-SOX9 (1:3000, Millipore, Billerica, MA, USA), anti-RUNX2, anti-P16 (1:3000, Abcam, Cambridge, UK), anti-COL2A1, anti-AGGRECAN, anti-OCT4, anti-MMP13, anti-COL1A1, anti-P21, anti-P53 (1:500, Santa Cruz Biotechnology, Santa Cruz, CA, USA), anti-NANOG (1:1000, BD biosciences, San Jose, CA, USA), anti-SOX2 (1:1000, Cell signaling Technology, Inc., Danver MA, USA), anti-HSP90 or anti-β-ACTIN (1:1,000, Santa Cruz Biotechnology) overnight at 4 °C. Then, membranes were incubated with secondary HRP-conjugated antibodies (1:5000, Santa Cruz Biotechnology) for 1 h at room temperature.

### Hydrogel precursor preparation

The hydrogel (Hy) was prepared as described previously^57,58^. In short, the cosolvent consisted of water and dimethylformamide at a ratio of 3:2; it was supplemented with 1-ethyl-3-(3-dimethylaminopropyl)-carbodiimide (EDC) and *N*-hydroxysuccinimide (NHS), which activates 3-(4-hydroxyphenyl)propionic acid (HPA), and this solution was added to a preheated gelatin solution. After 24 h of reaction at 40 °C, the solution was dialyzed with deionized water, filtered, and lyophilized.

### Animal experiments

Twenty New Zealand white rabbits (male, 3.5–4 kg; Doo Yeol Biotech, Seocho-gu, Seoul, Korea) were used to establish the osteochondral defect model according to previously methods^59^. Briefly, full-thickness cylindrical osteochondral defect sites with a diameter of 6 mm (3-mm depth) were formed on the surface of the femoral patellar groove using the osteochondral autotransfer system (OATS; Arthrex, Naples, FL, USA). For each osteochondral defect site, the following treatments were applied: None (defect), hydrogel only (Hy), Hy + P5-MSC (Hy/MSC), and Hy + P5-RMSC (Hy/RMSC). A total of 2 × 10^6^ in vitro cultured MSCs were mixed with the GH polymer (5 wt%) that containing 0.0015 mg/mL of horseradish peroxidase (HRP). The hydrogel formation was occurred when the solution mixed with same volume of GH polymer (5 wt%) that containing 0.005 wt% hydrogen peroxide (H_2_O_2_)^60^. At 8 weeks after the operation, the rabbits were euthanized and defect sites were extracted for the histological analysis. All animal experiments were approved by the Committee on the Ethics of Animal Experiments of Yonsei University College of Medicine (Permit No. 2016-0200).

### Histological evaluation

The samples obtained from osteochondral knee defects and micromass pellets were fixed for 7 days and 24 h in 10% formalin, respectively. After fixation, the specimens were embedded in paraffin and then paraffin sections were deparaffinized, rehydrated, and washed with PBS. The sections were stained with Masson’s trichrome (MT), hematoxylin–eosin (H&E), Safranin O (SafO), and Alcian blue (AB), as previously described^59^. The stained sections were observed using a VS 120 virtual microscope (Olympus, Tokyo, Japan). A quantitative analysis was performed using ImageJ v.1.48 (Aspire Software International, Leesburg, VA, USA). Moreover, the O’Drsicoll histological scoring system was used for the histological assessment^61^, and the regenerated cartilage was assessed by three blinded experts.

### Immunocytochemistry

After washing paraffin sections with PBS, they were incubated in hydrogen peroxidase for 10 min to minimize nonspecific background staining. To detect chondrogenic markers, the sections were incubated with anti-COL2A1, anti-AGGRECAN, and anti-COL10A1 (1:100, Santa Cruz Biotechnology) antibodies at 4 °C for at least 12 h. The attachment of secondary antibodies and fluorescent protein-conjugated secondary antibodies was performed following previously described methods^59^. Nuclei were stained with 4ʹ,6-diamidino-2-phenylindole (Sigma). An inverted fluorescence microscope (IX-71; Olympus, Tokyo, Japan) was used to acquire images and expression levels were quantified using ImageJ.

### Statistical analysis

Each experiment was performed in triplicate using samples from more than three donors. To detect differences between two groups, *t*-tests were used. The statistical significance of the differences among three or more groups was evaluated using one-way analysis of variance (ANOVA) with Tukey’s post hoc tests. All results are presented as means and 95% CIs of the values from three different donors per group.

## Electronic supplementary material

Supplementary Figure
