# Supplementary material for: Enhanced articular cartilage regeneration with SIRT1-activated MSCs using gelatin-based hydrogel
Source: Cell Death Dis. 2018 Aug 29;9(9):866. doi: 10.1038/s41419-018-0914-1 (PMC6115405; doi:10.1038/s41419-018-0914-1)
Supplement: Supplementary file 1 — Supplementary Figure [file 41419_2018_914_MOESM1_ESM.pptx]

## Slide 1
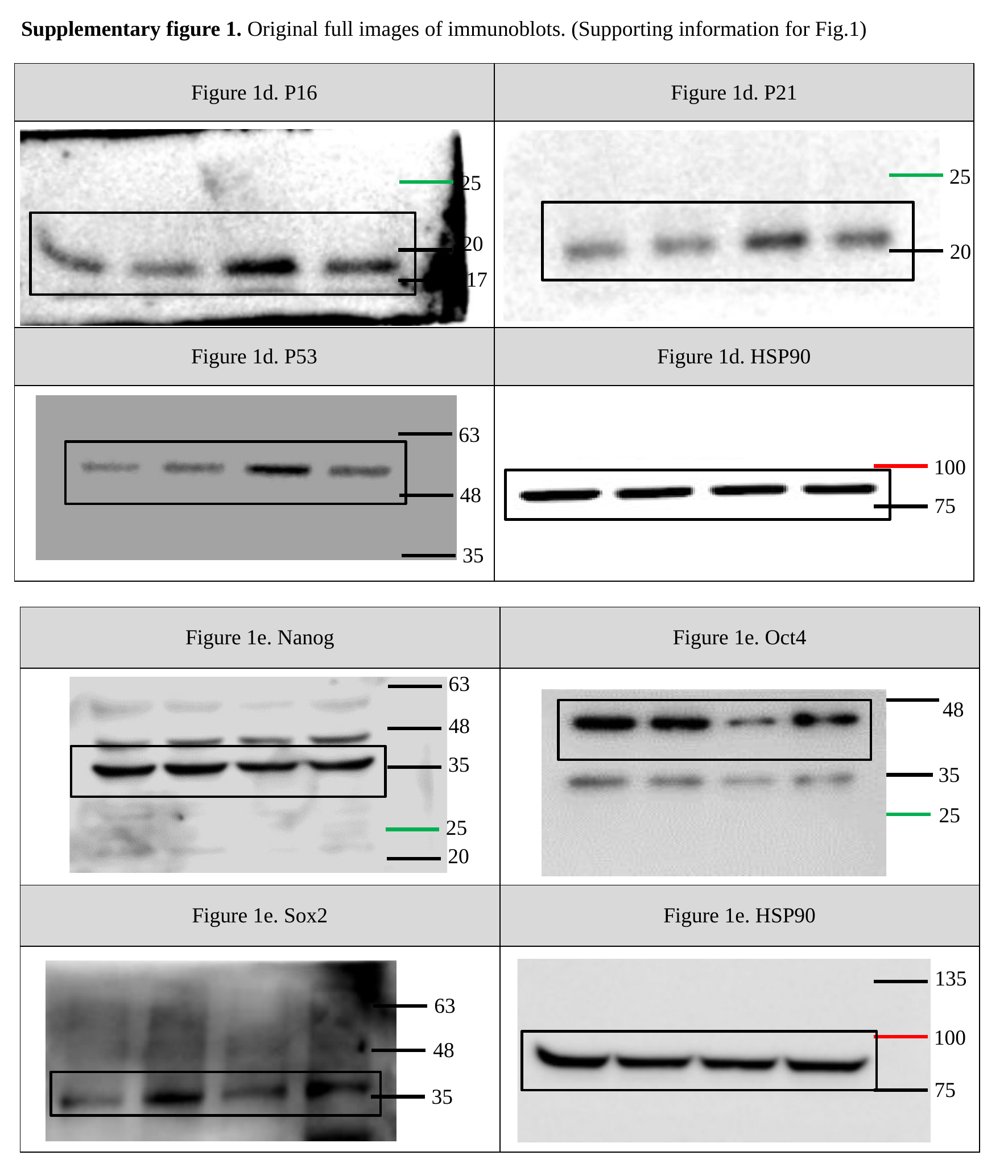

Supplementary figure 1. Original full images of immunoblots. (Supporting information for Fig.1)
| Figure 1d. P16 | Figure 1d. P21 |
| --- | --- |
| | |
| Figure 1d. P53 | Figure 1d. HSP90 |
| | |
25
25
20
20
17
63
100
48
75
35
| Figure 1e. Nanog | Figure 1e. Oct4 |
| --- | --- |
| | |
| Figure 1e. Sox2 | Figure 1e. HSP90 |
| | |
63
48
48
35
35
25
25
20
135
63
100
48
75
35

## Slide 2
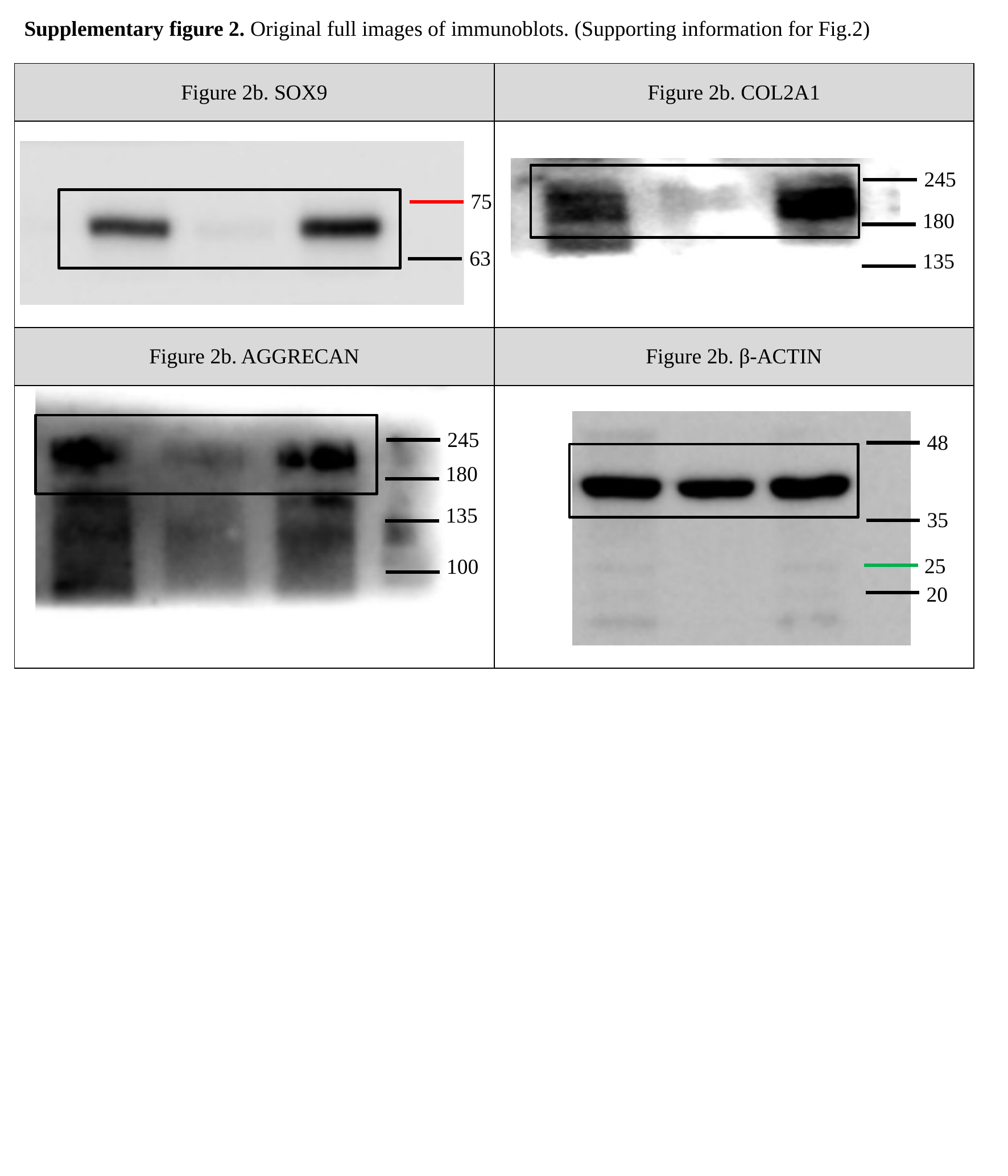

Supplementary figure 2. Original full images of immunoblots. (Supporting information for Fig.2)
| Figure 2b. SOX9 | Figure 2b. COL2A1 |
| --- | --- |
| | |
| Figure 2b. AGGRECAN | Figure 2b. β-ACTIN |
| | |
245
75
180
63
135
245
48
180
135
35
25
100
20

## Slide 3
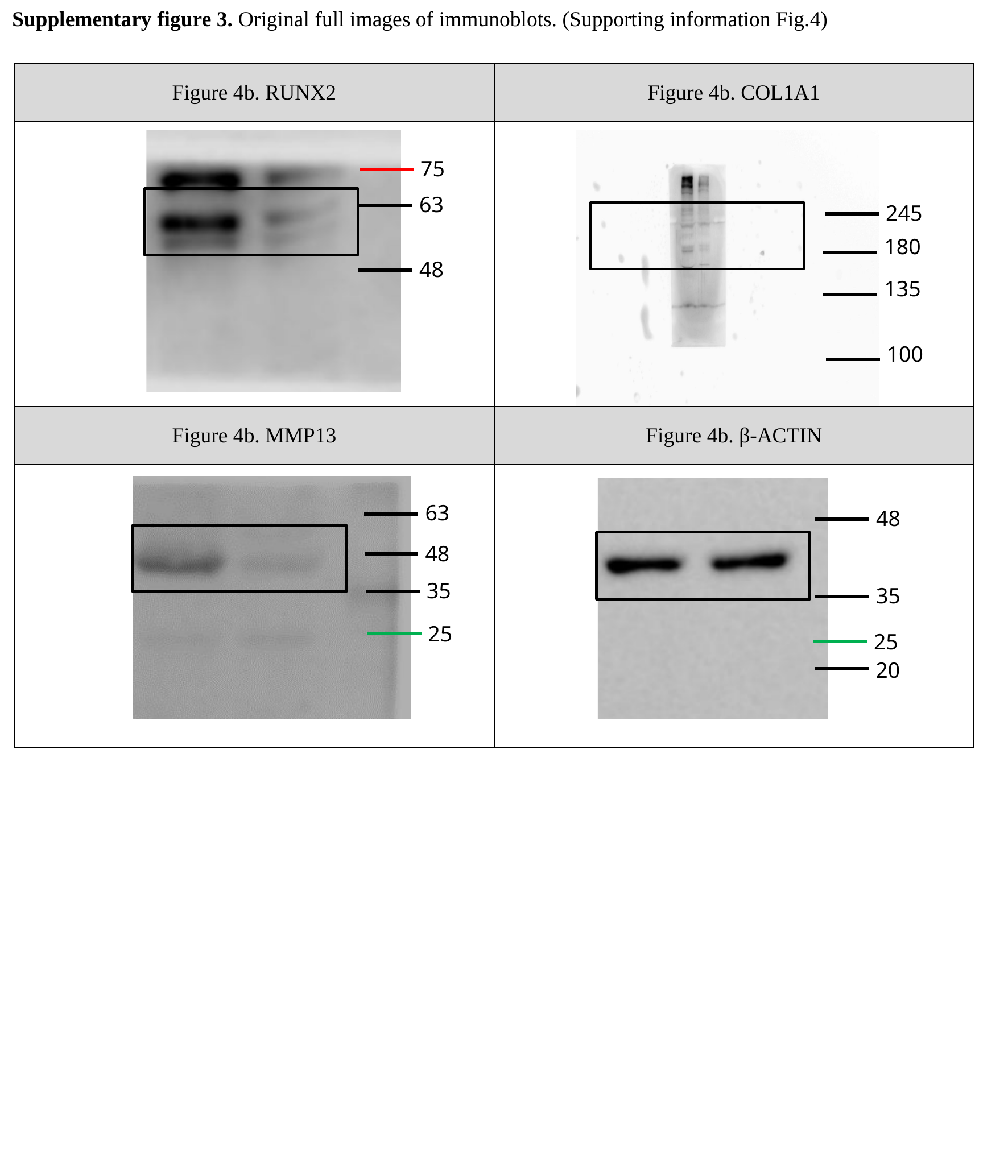

Supplementary figure 3. Original full images of immunoblots. (Supporting information Fig.4)
| Figure 4b. RUNX2 | Figure 4b. COL1A1 |
| --- | --- |
| | |
| Figure 4b. MMP13 | Figure 4b. β-ACTIN |
| | |
75
63
245
180
48
135
100
63
48
48
35
35
25
25
20
